# Micronutrient Dilution and Added Sugars Intake in U.S. Adults: Examining This Association Using NHANES 2009–2014

**DOI:** 10.3390/nu12040985

**Published:** 2020-04-02

**Authors:** Victor L. Fulgoni, P. Courtney Gaine, Maria O. Scott, Laurie Ricciuto, Loretta DiFrancesco

**Affiliations:** 1 Nutrition Impact, LLC, Battle Creek, MI 49014, USA; 2 The Sugar Association, Inc., Washington, DC 20005, USA; Gaine@sugar.org (P.C.G.); mscott@sugar.org (M.O.S.); 3 University of Toronto, Toronto, ON M4J 3E6, Canada; laurie.ricciuto@utoronto.ca; 4 Source! Nutrition, Toronto, ON M6S 5A6, Canada; loretta@sourcenutrition.com

**Keywords:** added sugars, micronutrient intake, micronutrient adequacy, adults, NHANES

## Abstract

There is inconsistent evidence regarding the impact of added sugars consumption on micronutrient dilution of the diet. We examined the associations between added sugars intake deciles and nutrient adequacy for 17 micronutrients in U.S. adults 19+ (*n* = 13,949), 19–50 (*n* = 7424), and 51+ y (*n* = 6525) using two days of 24 hour dietary recall data from the National Health and Nutrition Examination Survey (NHANES) 2009–2014 and regression analysis. Added sugars intake deciles ranged from <3.8 to >23.3% of calories among adults 19+ y, with a median intake of 11.0% of calories. Significant associations (*p* ≤ 0.01) between added sugars intake deciles and percentage of the population below the Estimated Average Requirement (EAR) were found for magnesium, vitamin C, vitamin D, and vitamin E; only the association with magnesium remained significant after dropping the two highest and lowest deciles of intake, suggesting a threshold effect. Intakes below approximately 18% of calories from added sugars were generally not associated with micronutrient inadequacy. However, even at the lower deciles of added sugars, large percentages of the population were below the EAR for these four micronutrients, suggesting that adequate intakes are difficult to achieve regardless of added sugars intake.

## 1. Introduction

Dietary guidelines in North America and worldwide call for restrictions on the intake of added sugars. The 2015–2020 Dietary Guidelines for Americans (DGA) recommend reducing added sugars intake to <10% of calories [1]. The World Health Organization recommends the same reduction on the intake of free sugars, but with a conditional recommendation for a further reduction to <5% of calories [2]. Governments have responded to this focus on sugars with nutrition labelling initiatives to help inform consumers about the added sugars content of foods. In 2016, the U.S. Food and Drug Administration issued a final rule on revisions to the Nutrition Facts label, including setting a maximum Daily Value (DV) of 50 g for added sugars, and mandatory declaration of the added sugars content of foods expressed in grams and as a percent of the DV [3]. Similarly, but with a focus on total sugars, Health Canada issued revised nutrition labelling regulations in 2016, including a DV of 100 g for total sugars [4].

Concerns about high added sugars intake, and hence the call for dietary restrictions, stem from the scientific literature on added sugars and health. The majority of the evidence is from studies of sugar-sweetened beverages, which have been shown to be associated with weight gain [5], type 2 diabetes [6], cardiometabolic risk factors, such as elevated blood pressure and lipids [7,8], and mortality from cardiovascular diseases [9]. Micronutrient dilution of the diet at higher added sugars intakes is also a concern; however, the evidence is not consistent and limited by methodological constraints [10] and/or has been studied mainly in children and adolescents [11,12,13].

Little is known about the potential for micronutrient dilution at higher added sugar intakes in the diet of the U.S. adult population. One study based on the National Health and Nutrition Examination Survey (NHANES) 2003–2006 has shown an inverse relationship with added sugars intake for some micronutrients [14]; however, there is a paucity of other evidence, most of which is from populations outside of North America [15,16,17] and/or on other measures of sugars intake, such as sugar-sweetened beverages [18] and free sugars [19]. It is also reasonable to speculate that micronutrient dilution of the diet might not be as evident in adults compared with children because adults have a higher calorie intake and a generally more varied food intake than children. Furthermore, some foods are more important sources of some nutrients for children than for adults, for example, milk is a key beverage for children to achieve their requisite calcium and vitamin D intakes [20].

We therefore examined associations between added sugars intake and micronutrient adequacy in U.S. adults, in whom there are different considerations than for children in terms of food intake and sources of added sugars and micronutrients. We analyzed these associations for 17 nutrients with an Estimated Average Requirement (EAR) across deciles of added sugars intake using NHANES 2009–2014.

## 2. Methods 

### 2.1. Data Source and Participants 

NHANES is a nationally-representative, cross-sectional survey conducted by the National Center for Health Statistics, part of the U.S. Centers for Disease Control and Prevention. An overview of NHANES, including the purpose, study population, sampling strategy, survey procedures, and response rates is provided elsewhere [21,22]. Briefly, NHANES dietary intake data were collected as part of *What We Eat in America* via two 24-h dietary recalls, the first collected in person in the health examination and the second conducted via telephone 3–10 days after the first. Both 24-h recalls were collected using the Automated Multiple-Pass Method [23]. For our analysis, we combined data from NHANES 2009–2014. The study protocol for NHANES was approved by the National Center for Health Statistics research ethics review board. Written informed consent was obtained for all participants [24]. 

### 2.2. Added Sugars and Micronutrient Intakes

Added sugars intake was determined using the USDA Food Patterns Equivalent Database (FPED) for each NHANES release [25]. The FPED defines added sugars as “sugars that are added to foods as an ingredient during preparation, processing, or at the table.” Added sugars do not include naturally occurring sugars, such as lactose present in milk and fructose present in whole or cut fruit, or 100% fruit juice; for FPED 2011–2012 and 2013–2014, but not FPED 2009–2010, fruit juice concentrates used as ingredients are also assigned to added sugars [25]. Intakes of added sugars in grams and calories were output, and percentage of total calories from added sugars was determined for each day of dietary recall. Subjects were then placed into deciles of added sugars intake as the average of the two days of recall as % of total calories; separate deciles were established for each age group (19+, 19–50, and 51+ y). Using a two-day average to define added sugars intake as a % of total calories was similar to the approach used in previously published work [14].

Intakes of 17 micronutrients with an EAR were obtained from NHANES dietary intake files [21]; these were calcium, copper, iron, magnesium, phosphorus, selenium, zinc, folate, niacin, riboflavin, thiamin, and vitamins A, B_12_, B_6_, C, D, and E. The National Cancer Institute (NCI) Method was used to estimate usual intake (UI) and distribution of intake of nutrients for each age group [26]. Given most micronutrients were consumed on most days by most subjects, the one-part model was used for the UI estimations. The two days of intake, using one-day sampling weights, were used to obtain percentiles of intake and necessary variance estimates. Covariates used in the NCI UI estimations were day of the week of the 24-h recall (coded as weekend (Friday to Sunday) or weekday (Monday to Thursday)) and sequence of dietary recall (first or second). Balanced repeated replication was performed to generate standard errors and balanced repeated replication weights were generated using Fay adjustment factor M = 0.3 with perturbation factor of 0.7, which were then adjusted to match initial sample weights within age, gender, and race/ethnicity groups. Given the EAR is the appropriate Dietary Reference Intake to use when assessing the adequacy of population micronutrient intakes [27], the EAR cut-point method was used to estimate the percentage of an age group with intakes below requirements (prevalence of inadequacy) within each decile of added sugars intake. However, to determine the prevalence of inadequate intake of iron, the probability method was used [27]. Micronutrient contributions from dietary supplements were not included because the primary research question examined intakes from foods and beverages.

### 2.3. Statistical Analysis

Linear regression analyses were conducted within the three age groups (19+, 19–50, and 51+ y) to determine whether relationships existed between decile of added sugars intake and micronutrient adequacy. Dummy-coded deciles of intake (1–10) were used as the independent variable. Regression coefficients were generated to examine the magnitude of significant relationships and showed the change in percentage of the population with inadequate intakes (below the EAR) as added sugars intake increased across deciles of intake. Sensitivity analysis was performed to examine the influence of the extremes of intakes of added sugars to the main findings, first by restricting the analysis to deciles two through nine, and second by restricting the analysis to deciles three through eight. Additionally, to assess whether the relationship of added sugars intake with prevalence of micronutrient inadequacy was curvilinear, regression analyses were conducted using the linear and quadratic term of dummy variable for decile number; if the quadratic term was significant, then a curvilinear relationship was present. Statistical significance was set at *p* ≤ 0.01 (however, for those who wish to use a different *p*-value e.g., *p* ≤ 0.05 or a Bonferroni adjustment, we present actual *p*-values for all trends evaluated).

## 3. Results

After excluding those with unreliable dietary records (*n* = 61) as determined by USDA National Center for Health Statistics staff, those pregnant or lactating (*n* = 240), those with zero calories (*n* = 16), and those with only a single dietary recall (*n* = 1867), the final sample was 13,949 (6746 males and 7203 females), with 7424 (3600 males and 3824 females) participants 19–50 y and 6525 (3146 males and 3379 females) participants 51+ y. The average age (standard error) of those 19+ y was 47.1 (0.4) y, and the average age of those 19–50 and 51+ y was 34.6 (0.3) and 63.6 (0.2) y, respectively.

### 3.1. Added Sugars Intakes

The average of two dietary recalls of added sugars intake (percentage of calories) across deciles of intake ranged from <3.8 to >23.3 in adults aged 19+ y (Table 1). Median added sugars intake was 11.0% of calories, and was higher among younger adults 19–50 y of age (11.8%) and lower among older adults aged 51+ y (10.1%).

### 3.2. Relationship of Added Sugars Intake to Micronutrient Adequacy

Among adults aged 19+ y, there was a significant association between added sugars intake and micronutrient inadequacy for four of the 17 micronutrients we examined: magnesium (β ± SE: 4.0 ± 1.0 percentage units per decile), vitamin C (2.4 ± 0.7 percentage units per decile), vitamin D (0.7 ± 0.09 percentage units per decile), and vitamin E (2.0 ± 0.3 percentage units per decile) (Table 2). For example, for magnesium, each one-decile increase in added sugars intake was associated with an increase of 4.0 percentage units of adults with inadequate magnesium intakes (below the EAR). The association for magnesium was also significant among younger adults aged 19–50 y (5.5 ± 1.0 percentage units per decile), but did not reach significance among older adults (51+ y). In younger adults aged 19–50 y, a significant relationship with added sugars intake was found for magnesium (5.5 ± 1.0 percentage units per decile), and vitamins A (3.4 ± 0.9 percentage units per decile), C (3.4 ± 0.6 percentage units per decile), D (0.7 ± 0.1 percentage units per decile), and E (2.5 ± 0.3 percentage units per decile); however, in older adults aged 51+ y, only the relationship for vitamin D (0.5 ± 0.1 percentage units per decile) was significant (Table 2).

When linear regression analyses were repeated with the lowest and highest deciles dropped (1 and 10), the positive associations between added sugars intake and percentage of the population below the EAR persisted for both magnesium and vitamin E in adults 19+ y (Table 3). In those 19–50 y, there were significant relationships for magnesium, folate, thiamin, and vitamins A, C, and E; there were no significant relationships in those 51+ y after removing the highest and lowest deciles of intake (Table 3). When two more of the deciles at the tail ends were dropped (1, 2 and 9, 10), there were significant associations between added sugars intake and micronutrient adequacy for magnesium, folate, and vitamin A in those 19+ y (Table 4), which were driven by results in the 19–50 y age group, as again there were no significant associations of added sugars intake with micronutrient adequacy in those 51+ y. In the 19–50 y age group, a significant relationship was also found for vitamin E.

**Figure 1:**
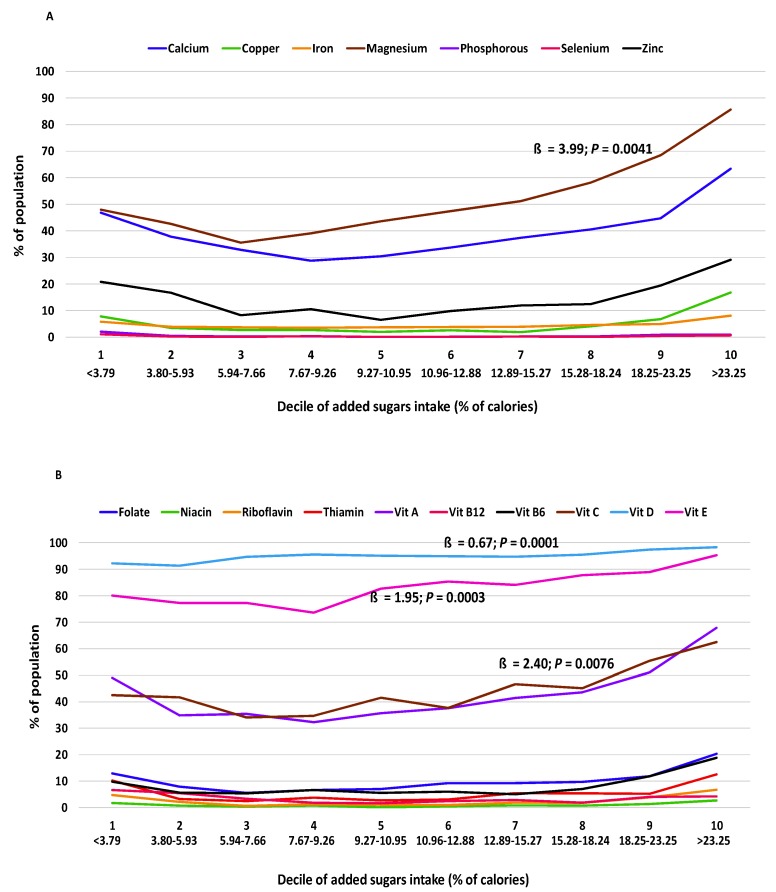
Percentage of adults 19+ y (NHANES 2009–2014) with micronutrient intakes below the EAR for (**A**) minerals and (**B**) vitamins per decile of added sugars intake (% of calories) averaged across two days of intake using regression analysis (ß: regression coefficient). Association deemed significant at *p* < 0.01. EAR, Estimated Average Requirement; Vit, vitamin.

**Figure 2:**
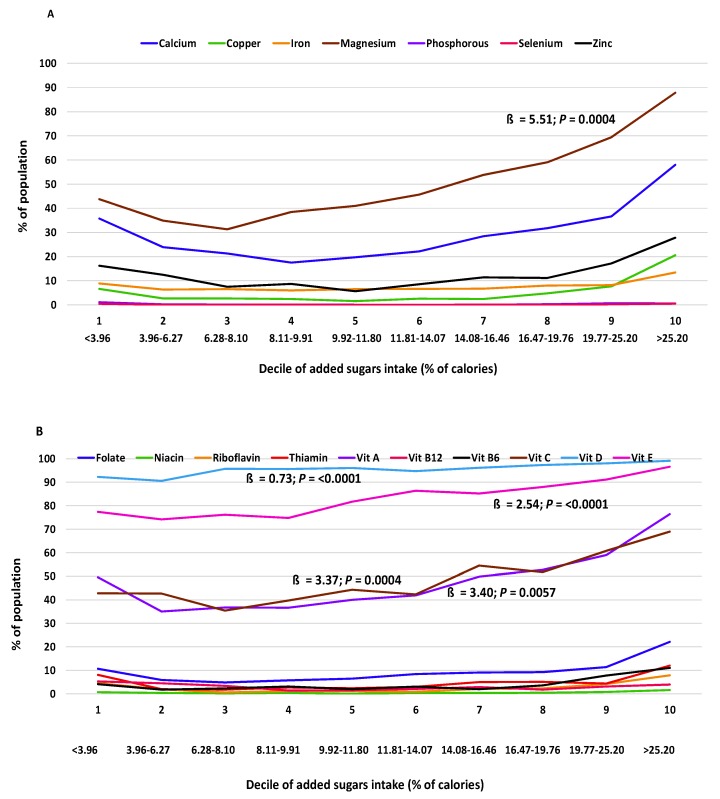
Percentage of adults 19–50 y (NHANES 2009–2014) with micronutrient intakes below the EAR for (**A**) minerals and (**B**) vitamins per decile of added sugars intake (% of calories) averaged across two days of intake using regression analysis (ß: regression coefficient). Association deemed significant at *p* < 0.01. EAR, Estimated Average Requirement; Vit, vitamin.

**Figure 3:**
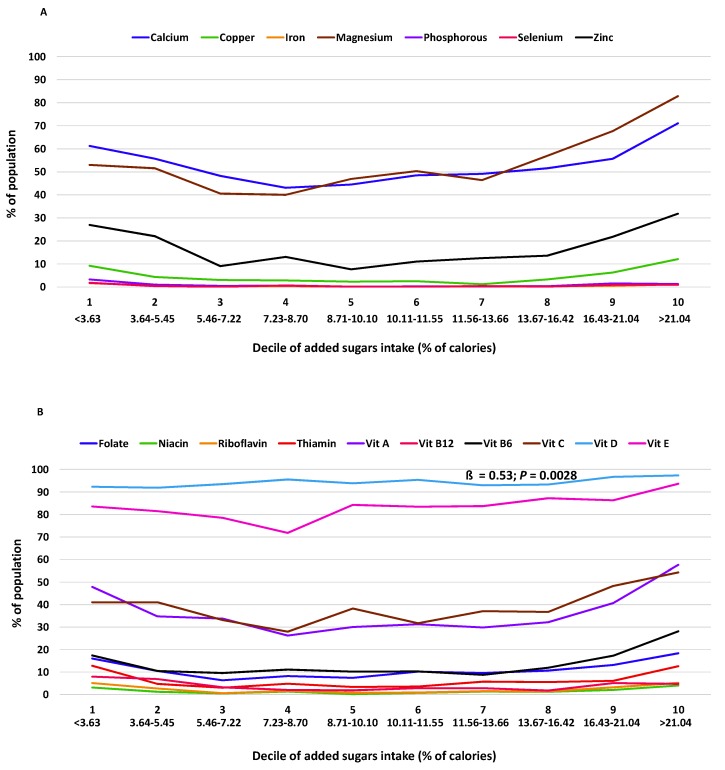
Percentage of adults 51+ y (NHANES 2009–2014) with micronutrient intakes below the EAR for (**A**) minerals and (**B**) vitamins per decile of added sugars intake (% of calories) averaged across two days of intake using regression analysis (ß: regression coefficient). Association deemed significant at *p* < 0.01. EAR, Estimate.

The relationship of added sugars intake with micronutrient adequacy was curvilinear for 12 of 17 nutrients examined (calcium, copper, iron, magnesium, zinc, folate, niacin, riboflavin, and vitamins A, B_12_, B_6_, and C) in adults 19+ y, indicating that as added sugars increased, the degree of micronutrient inadequacy increased at a faster rate (Table 5). Curvilinear shapes of the relationship can also be seen in the presentation of results from the regression analyses across the added sugars deciles (Figure 1, Figure 2 and Figure 3). 

## 4. Discussion

Our results across deciles of added sugars intake among U.S. adults using NHANES 2009–2014 provide recent estimates of added sugars intakes for this population. Median intake of added sugars was 11.0% of calories among adults 19+ y; and intake was higher among younger adults (11.8%) and lower among older adults (10.1%), which is a demographic trend also seen in previous surveys in the United States [14,28], and in national surveys in Australia and Sweden [19,29]. Our results of added sugars consumption among adults are similar to an analysis of NHANES 2011–2012 data, which reported an average added sugars intake of 14% of calories [30]. In other countries, lower added sugars intakes have been reported [31] and reductions in sugar-sweetened beverage consumption have been documented [18]. As the food landscape changes in response to dietary guidance on reducing added sugars intake, along with new nutrition labels requiring the declaration of the added sugars content of foods, trends in added sugars intake and current food sources of added sugars should be continually monitored.

Our estimate of median added sugars intake of 11.0% of calories indicates that half of U.S. adults were either below or very close to the DGA recommendation of <10% of calories from added sugars [1]. It also indicates that half of the population was above the recommendation, which raises questions about the potential for micronutrient dilution of the diet of U.S. adults, given the DGA recommendation is partly based on this concern. Of the 17 micronutrients we examined, magnesium, and vitamins C, D, and E consistently showed significant increases in the percentage of adults with inadequate intakes (below the EAR) as added sugars intake increased. Significant associations with added sugars intake were also apparent for another two of the micronutrients we studied (vitamin A and zinc) for at least one age group. While direct comparisons with other studies are limited due to methodological differences, our findings are consistent with other research, showing magnesium and vitamin C inadequacies increase as added sugars intakes increase [14,16,18,19]. Given that our results indicate added sugars intake appeared to impact four of the 17 micronutrients we examined in all adults (and six of 17 micronutrients in those 19–50 y), increased added sugars intake may be one of many dietary factors associated with micronutrient adequacy in adults.

The types of foods and beverages contributing to added sugars consumption at different intake levels may partly explain the associations we found. A recent study has shown that among adults consuming the highest amount of added sugars (decile 10), sweetened beverages, coffee, and tea together accounted for 62% of their added sugars intake [32]. The lack of nutrients (such as magnesium) in sugar-sweetened beverages and the absence of nutrients in other beverages (coffee and tea) could thus explain the higher micronutrient inadequacies that we found across higher deciles of added sugars intake. In the same study, the top sources of added sugars among adults consuming the lowest amount of added sugars (decile 1) were breads/rolls/tortillas, sweet bakery products, and ready-to-eat cereals (RTEC), together accounting for 38% of their added sugars intake [32]. Magnesium from grain-based products (such as breads and RTEC) could thus explain the lower magnesium inadequacies that we observed across lower deciles of added sugars intake. Furthermore, it is possible intakes of foods and beverages such as milk, fruit, and juice, which tend to be consumed along with those grain-based products, could also explain the lower inadequacies of magnesium and vitamin C that we found across lower deciles of added sugars intake. Taken together, these findings illustrate the complexity of the relationship between added sugars and micronutrient adequacy, as not all foods higher in added sugars compromise micronutrient intakes to the same extent; some such foods may even help to ameliorate inadequate intakes.

Further elucidation of the impact of added sugars intake on micronutrient adequacy can be gleaned from our truncated analyses (running regressions after dropping extremes in added sugars intake). When we repeated linear regressions across added sugars deciles excluding the two lowest and highest deciles, associations for three of the micronutrients (magnesium, folate, and vitamin A) were apparent among adults 19+ y; and an association with vitamin E was apparent among younger adults (19–50 y). Together with our evaluation of curvilinear relationships of added sugars intake and micronutrient adequacy, these results suggest the impact of added sugars on micronutrient adequacy may only be an issue at the highest levels of added sugars intake (>18% of calories). This interpretation is consistent with other research showing a threshold effect, whereby micronutrient inadequacies were found to increase significantly only above a certain level of added sugars intake (20%–25% of calories) [14,19]. A greater consumption of sugar-sweetened beverages among the highest consumers of added sugars [32] might contribute to this threshold effect insofar as these beverages are not substantive sources of micronutrients.

Magnesium, and vitamins C, D, and E are among the micronutrients that have been characterized as shortfall nutrients in the U.S. diet [33]. Several reports have also stressed that adequate intake of one or more of these nutrients is especially important for older populations [34,35]. While we observed an increase in the prevalence of inadequacy for these micronutrients as added sugars increased, substantial percentages of the population were below the EAR even at the lower deciles of added sugars intake. Low intakes of dairy, vegetables, fruit, and whole grains, which are important sources of magnesium and vitamin C, have been reported among U.S. adults regardless of added sugars intake [1], lending support to our findings of inadequacy across the range of added sugars intakes. Given current dietary patterns, it appears that adequate intakes of magnesium and vitamin C are difficult for adults to achieve independent of added sugars intake.

To achieve nutrient adequacy and help prevent chronic disease, the DGA emphasize “healthy eating patterns” [1]. Studies examining eating patterns among adults portray interrelationships among various aspects of the diet. In one study, researchers have found that snacking had a greater impact on total sugar intake than meals, such that a higher frequency of snacking was associated with significantly higher total sugars intakes [36]. Results from another study have shown a higher consumption of sugar-sweetened beverages was associated with lower fruit and vegetable consumption [18]; while results from an intervention study have shown that a singular focus on reducing the consumption of sugar-sweetened beverages not only achieved significant reductions in the consumption of those beverages, but also improved other aspects of the diet, including reduced intakes of energy, trans fat, and sugars [37]. It is therefore important to consider overall eating patterns when making dietary recommendations and implementing interventions focused on added sugars.

Our study has some strengths and limitations. Using NHANES data from three cycles provided a large amount of data collected under commonly accepted procedures. Regression analyses using NHANES weights and design criteria allowed us to estimate the magnitude of change in the percentage of the population below the EAR as added sugars intake increased. Furthermore, we used deciles of added sugars intake versus arbitrarily selected set points, allowing us to examine points along the continuum of added sugars intake where micronutrient intakes are sufficient and/or begin to be a concern. In order to minimize misclassification errors, we used the two-day average intake of added sugars rather than one-day intakes or modeling intake (i.e., UI) to define the added sugars deciles. We also ran regression analyses with added sugars intake entered as either a decile variable (values from one to 10) or as an actual intake variable (% of calories), and consistencies in the results of these regressions confirmed that misclassification was unlikely to impact our results dramatically.

As for limitations, even using a two-day average of added sugars intake rather than a measure of long-term habitual intake may lead to biased estimates of added sugars intake. The error in the added sugars intake variable due to within-person variation would also cause an attenuation of any affect, meaning we would be less likely to see associations between added sugars intake and micronutrient adequacy. When we conducted the same analyses with UI to define added sugars intake, the results were similar (added sugars intakes were only associated with the few nutrients described above, data not shown), suggesting our analyses using a two-day average of added sugars intake did not limit our detection of relationships. 

Other limitations include dietary intake data that are self-reported, and while the best procedures were used to assess micronutrient intake, self-reported dietary data rely on memory and the ability of subjects to accurately report the foods and amounts of foods consumed. It is also unknown if there is an under-reporting bias regarding foods with added sugars; if such under- reporting exists, it would impact our added sugars intake estimates.

## 5. Conclusions

In conclusion, using NHANES 2009–2014, we found that the median intake of added sugars among U.S. adults was 11.0% of calories, suggesting that half of the population was either below or very close to the DGA limit of <10% of calories from added sugars [1]. Higher added sugars intake was significantly associated with higher prevalence of inadequacy in four of the 17 micronutrients we examined (magnesium, and vitamins C, D, and E), with some variation among older and younger adults, and an apparent threshold effect (<18% of calories). However, there were large percentages of the population with intakes below the EAR for these micronutrients, even at the lower deciles of added sugars intake, suggesting that adequate intakes of these nutrients are difficult for adults to achieve within their current dietary patterns, particularly for younger adults. Our findings also suggest that recommending an added sugars limit of <10% of calories, in and of itself, may not enhance diet quality and micronutrient adequacy across the population without further recommendations on which specific foods with added sugars to decrease. It might be more effective to target dietary guidelines on added sugars to specific population subgroups, considering their overall diet quality, micronutrient adequacy, and specific food and beverage sources of added sugars. As public policy moves forward with dietary recommendations and nutrition labeling focused on added sugars, it will be important to monitor dietary patterns and diet quality across various population subgroups in order to evaluate the impact of these interventions. Our study also provides a benchmark for future dietary shifts as dietary guidance on added sugars progresses and alongside any resultant changes in the food supply.

## Figures and Tables

**Table 1:** Deciles of two-day average intake of added sugars (% of calories) ^1,2^ in adults 19+ y (National Health and Nutrition Examination Survey (NHANES) 2009–2014).

Age Group	D ^3^1	D2	D3	D4	D5	D6	D7	D8	D9	D10
19+ y	<3.79	≥3.79–≤5.93	>5.93–≤7.66	>7.66–≤9.26	>9.26–≤10.95	>10.95–≤12.88	>12.88–≤15.27	>15.27–≤18.24	>18.24–≤23.25	>23.25
19–50 y	<3.96	≥3.96–≤6.27	>6.27–≤8.10	>8.10–≤9.91	>9.91–≤11.80	>11.80–≤14.07	>14.07–≤16.46	>16.46–≤19.76	>19.76–≤25.20	>25.20
51+ y	<3.63	≥3.63–≤5.45	>5.45–≤7.22	>7.22–≤8.70	>8.70‒≤10.10	>10.10–≤11.55	>11.55–≤13.66	>13.66–≤16.42	>16.42–≤21.04	>21.04

^1^ Calculated as the average of each dietary recall taking 100× ratio of intake of added sugars (kcal) to intake of total calories (kcal) averaged across two days of intake. ^2^ Data source for added sugars: USDA Food Patterns Equivalent Database 2009–2010, 2011–2012, and 2013–2014. ^3^ D, decile.

**Table 2:** Association between added sugars intake (% of calories) and percentage of adults 19+ y (NHANES 2009–2014) with micronutrient intakes below the EAR ^1^.

	Age Group					
	19+ y		19–50 y		51+ y	
	ß * ± SE	*p* **	ß ± SE	*p*	ß ± SE	*p*
Minerals						
Calcium	1.47 ± 0.98	0.1727	2.42 ± 1.10	0.0589	0.72 ± 0.94	0.4675
Copper	0.27 ± 0.37	0.4969	0.33 ± 0.35	0.3735	0.13 ± 0.36	0.7287
Iron	0.16 ± 0.12	0.2282	0.33 ± 0.19	0.1234	−0.01 ± 0.03	0.7076
Magnesium	3.99 ± 1.00	**0.0041**	5.51 ± 0.95	**0.0004**	2.92 ± 1.12	0.0310
Phosphorus	−0.02 ± 0.05	0.6471	0.0007 ± 0.0274	0.9790	−0.04 ± 0.07	0.5540
Selenium	0.003 ± 0.022	0.8828	−0.001 ± 0.009	0.8945	0.01 ± 0.04	0.8238
Zinc	0.51 ± 0.72	0.4978	0.56 ± 0.59	0.3634	0.35 ± 0.96	0.7285
Vitamins						
Folate	0.53 ± 0.36	0.1760	0.89 ± 0.34	0.0310	0.26 ± 0.42	0.5451
Niacin	0.005 ± 0.087	0.9569	0.02 ± 0.05	0.6968	0.004 ± 0.146	0.9786
Riboflavin	0.18 ± 0.16	0.2747	0.23 ± 0.17	0.2076	0.10 ± 0.15	0.5313
Thiamin	0.29 ± 0.27	0.3115	0.41 ± 0.20	0.0729	0.13 ± 0.35	0.7182
Vitamin A	1.82 ± 0.92	0.0828	3.40 ± 0.91	**0.0057**	0.43 ± 1.00	0.6793
Vitamin B_12_	−0.23 ± 0.19	0.2661	−0.19 ± 0.16	0.2681	−0.27 ± 0.22	0.2532
Vitamin B_6_	0.28 ± 0.34	0.4460	0.22 ± 0.18	0.2576	0.56 ± 0.57	0.3490
Vitamin C	2.40 ± 0.68	**0.0076**	3.37 ± 0.59	**0.0004**	1.10 ± 0.79	0.2052
Vitamin D	0.67 ± 0.09	**0.0001**	0.73 ± 0.09	**<0.0001**	0.53 ± 0.12	**0.0028**
Vitamin E	1.95 ± 0.32	**0.0003**	2.54 ± 0.26	**<0.0001**	1.23 ± 0.38	0.0122

^1^ EAR, Estimated Average Requirement. * Regression coefficient (ß) across deciles of intake of added sugars averaged across two days of intake. ** *p*-value for estimating the probability of rejecting the null hypothesis that the slope of the association is zero (ß = 0) when it is true; *p* ≤ 0.01 deemed significant (bolded values).

**Table 3:** Association between added sugars intake (% of calories) and percentage of adults 19+ y (NHANES 2009–2014) with micronutrient intakes below the EAR ^1^ using deciles 2–9.

	Age Group					
	19+ y		19–50 y		51+ y	
	ß * ± SE	*p* **	ß ± SE	*p*	ß ± SE	*p*
Minerals						
Calcium	1.54 ± 0.62	0.0477	1.96 ± 0.65	0.0243	0.52 ± 0.72	0.4989
Copper	0.16 ± 0.22	0.4860	0.18 ± 0.26	0.5364	−0.04 ± 0.24	0.8713
Iron	0.12 ± 0.05	0.0608	0.25 ± 0.07	0.0147	−0.01 ± 0.02	0.6135
Magnesium	3.35 ± 0.82	**0.0064**	4.93 ± 0.60	**0.0002**	1.70 ± 1.16	0.1921
Phosphorus	−0.004 ± 0.034	0.9100	0.01 ± 0.02	0.6995	−0.03 ± 0.05	0.6492
Selenium	0.01 ± 0.02	0.5533	0.0005 ± 0.0076	0.9544	0.02 ± 0.03	0.5330
Zinc	0.64 ± 0.67	0.3739	0.64 ± 0.53	0.2768	0.31 ± 0.92	0.7473
Vitamins						
Folate	0.55 ± 0.21	0.0363	0.76 ± 0.14	**0.0015**	0.47 ± 0.31	0.1810
Niacin	0.03 ± 0.08	0. 6940	0.03 ± 0.05	0.5197	0.05 ± 0.14	0.7279
Riboflavin	0.19 ± 0.12	0.1753	0.26 ± 0.12	0. 0743	0.11 ± 0.13	0.4354
Thiamin	0.39 ± 0.14	0.0305	0.43 ± 0.09	**0.0028**	0.31 ± 0.18	0.1249
Vitamin A	2.11 ± 0.43	0.0027	3.34 ± 0.37	**0.0001**	0.42 ± 0.62	0.5230
Vitamin B_12_	−0.11 ± 0.21	0.6003	−0.12 ± 0.18	0.5171	−0.16 ± 0.22	0.5098
Vitamin B_6_	0.29 ± 0.22	0.2467	0.23 ± 0.15	0.1618	0.41 ± 0.31	0.2255
Vitamin C	1.98 ± 0.76	0.0393	2.94 ± 0.70	**0.0056**	0.92 ± 0.98	0.3816
Vitamin D	0.54 ± 0.17	0.0177	0.64 ± 0.18	0.0126	0.37 ± 0.23	0.1533
Vitamin E	1.89 ± 0.36	**0.0021**	2.53 ± 0.26	**0.0001**	1.01 ± 0.49	0.0858

^1^ EAR, Estimated Average Requirement. * Regression coefficient (ß) across deciles of added sugars averaged across two days of intake. ** *p*-value for estimating the probability of rejecting the null hypothesis that the slope of the association is zero (ß = 0) when it is true; *p* ≤ 0.01 deemed significant (bolded values).

**Table 4:** Association between added sugars intake (% of calories) and percentage of adults 19+ y (NHANES 2009–2014) with micronutrient intakes below the EAR ^1^ using deciles 3–8.

	Age Group					
	19+ y		19–50 y		51+ y	
	ß * ± SE	*p* **	ß ± SE	*p*	ß ± SE	*p*
Minerals						
Calcium	1.72 ± 0.64	0.0535	2.67 ± 0.74	0.0203	0.95 ± 0.63	0.2083
Copper	0.09 ± 0.20	0.6852	0.18 ± 0.26	0.5364	−0.13 ± 0.21	0.5653
Iron	0.15 ± 0.06	0.0617	0.26 ± 0.12	0.0958	−0.002 ± 0.004	0.6086
Magnesium	4.34 ± 0.24	**0.0001**	5.45 ± 0.35	**0.0001**	3.03 ± 0.79	0.0185
Phosphorus	0.001 ± 0.025	0.9706	0.01 ± 0.02	0.5784	−0.02 ± 0.04	0.5505
Selenium	0.01 ± 0.01	0.6101	−0.001 ± 0.009	0.8945	0.01 ± 0.02	0.5915
Zinc	0.70 ± 0.34	0.1102	0.75 ± 0.41	0.1414	0.72 ± 0.42	0.1566
Vitamins						
Folate	0.91 ± 0.14	**0.0030**	0.99 ± 0.14	**0.0020**	0.82 ± 0.20	0.0148
Niacin	0.07 ± 0.10	0. 5614	0.05 ± 0.06	0.4361	0.13 ± 0.17	0.5016
Riboflavin	0.23 ± 0.06	0.0225	0.31 ± 0.08	0.9183	0.14 ± 0.07	0.1236
Thiamin	0.55 ± 0.22	0.0716	0.64 ± 0.16	0.0729	0.45 ± 0.22	0.1064
Vitamin A	1.98 ± 0.41	**0.0088**	3.51 ± 0.51	**0.0023**	−0.14 ± 0.60	0.8213
Vitamin B_12_	−0.06 ± 0.17	0.7375	0.003 ± 0.193	0.9868	−0.16 ± 0.14	0.3151
Vitamin B_6_	0.05 ± 0.19	0.8202	0.02 ± 0.16	0.9255	0.10 ± 0.26	0.7119
Vitamin C	2.32 ± 0.79	0.0423	3.86 ± 0.88	0.0119	0.88 ± 1.01	0.4329
Vitamin D	0.05 ± 0.09	0.6351	0.27 ± 0.16	0.1639	−0.25 ± 0.28	0.4196
Vitamin E	2.40 ± 0.61	0.0167	2.71 ± 0.50	**0.0057**	2.00 ± 0.78	0.0616

^1^ EAR, Estimated Average Requirement. * Regression coefficient (ß) across deciles of added sugars averaged across two days of intake. ** *p*-value for estimating the probability of rejecting the null hypothesis that the slope of the association is zero (ß = 0) when it is true; *p* ≤ 0.01 deemed significant (bolded values).

**Table 5:** Curvilinear association between added sugars intake (% of calories) and percentage of adults 19+ y (NHANES 2009–2014) with micronutrient intakes below the EAR ^1^ across sugar intake deciles.

	Age Group					
	19+ y		19–50 y		51+ y	
	ß * ± SE	*p* **	ß ± SE	*p*	ß ± SE	*p*
Minerals						
Calcium	1.10 ± 0.13	**0.0001**	1.19 ± 0.15	**0.0001**	1.00 ± 0.11	**<0.0001**
Copper	0.37 ± 0.08	**0.0020**	0.36 ± 0.08	**0.0026**	0.37 ± 0.07	**0.0012**
Iron	0.12 ± 0.03	**0.0028**	0.18 ± 0.05	**0.0065**	0.03 ± 0.01	0.0291
Magnesium	1.12 ± 0.10	**<0.0001**	1.09 ± 0.13	**0.0001**	1.18 ± 0.16	**0.0002**
Phosphorus	0.04 ± 0.01	0.0131	0.03 ± 0.01	**0.0035**	0.07 ± 0.02	0.0101
Selenium	0.02 ± 0.01	0.2077	0.008 ± 0.004	0.0991	0.03 ± 0.02	0.1485
Zinc	0.81 ± 0.12	**0.0003**	0.66 ± 0.08	**0.0001**	1.03 ± 0.18	**0.0007**
Vitamins						
Folate	0.35 ± 0.08	**0.0040**	0.31 ± 0.09	0.0124	0.42 ± 0.09	**0.0022**
Niacin	0.08 ± 0.02	**0.0014**	0.04 ± 0.01	**0.0035**	0.14 ± 0.03	**0.0022**
Riboflavin	0.17 ± 0.05	**0.0071**	0.19 ± 0.04	**0.0023**	0.15 ± 0.05	0.0185
Thiamin	0.22 ± 0.09	0.0450	0.13 ± 0.08	0.1503	0.31 ± 0.10	0.0155
Vitamin A	1.07 ± 0.16	**0.0003**	1.03 ± 0.17	**0.0005**	1.12 ± 0.16	**0.0002**
Vitamin B_12_	0.19 ± 0.04	**0.0027**	0.15 ± 0.04	**0.0049**	0.23 ± 0.06	**0.0056**
Vitamin B_6_	0.35 ± 0.09	**0.0048**	0.18 ± 0.07	0.0431	0.57 ± 0.11	**0.0013**
Vitamin C	0.64 ± 0.12	**0.0012**	0.52 ± 0.13	**0.0061**	0.75 ± 0.15	**0.0017**
Vitamin D	0.03 ± 0.04	0.5328	0.004 ± 0.042	0.9265	0.04 ± 0.05	0.5283
Vitamin E	0.32 ± 0.09	0.0107	0.27 ± 0.08	0.0102	0.34 ± 0.12	0.0250

^1^ EAR, Estimated Average Requirement. * Regression coefficient (ß) for quadratic component of regression analyses across deciles of added sugars averaged across two days of intake. ** *p*-value for estimating the probability of rejecting the null hypothesis that the slope of the association is zero (ß = 0) when it is true; *p* ≤ 0.01 deemed significant (bolded values).
